# Korean Pine Nut Oil Attenuated Hepatic Triacylglycerol Accumulation in High-Fat Diet-Induced Obese Mice

**DOI:** 10.3390/nu8010059

**Published:** 2016-01-21

**Authors:** Soyoung Park, Sunhye Shin, Yeseo Lim, Jae Hoon Shin, Je Kyung Seong, Sung Nim Han

**Affiliations:** 1 Department of Food and Nutrition, College of Human Ecology, Seoul National University, Seoul 151-742, Korea; naroo24@snu.ac.kr (S.P.); shanhui1@snu.ac.kr (S.S.); mee1132@snu.ac.kr (Y.L.); 2 College of Veterinary Medicine, BK21 Program for Veterinary Science, Seoul National University, Seoul 151-742, Korea; water21@snu.ac.kr (J.H.S.); snumouse@snu.ac.kr (J.K.S.); 3 Research Institute for Veterinary Science, Seoul National University, Seoul 151-742, Korea; 4 Bio-MAX Institute, Seoul National University, Seoul 151-742, Korea; 5 Research Institute of Human Ecology, Seoul National University, Seoul 151-742, Korea

**Keywords:** pine nut oil, high-fat diet, obesity, hepatic steatosis, SIRT3

## Abstract

Korean pine nut oil (PNO) has been reported to influence weight gain and lipid metabolism. We examined whether PNO replacement in a high-fat diet (HFD) can ameliorate HFD-induced hepatic steatosis. Five-week-old male C57BL mice were fed control diets containing 10% of the energy from fat from PNO or soybean oil (SBO) (PC, SC) or HFDs with 45% of the energy from fat, with 10% from PNO or SBO and 35% from lard (PHFD, SHFD), for 12 weeks. Body weight gain and amount of white adipose tissue were lower in PHFD (10% and 18% lower, respectively) compared with SHFD. Hepatic triacylglycerol (TG) level was significantly lower in PHFD than the SHFD (26% lower). PNO consumption upregulated hepatic ACADL mRNA levels. The hepatic PPARG mRNA level was lower in the PC than in the SC. Expression of the sirtuin (SIRT) 3 protein in white adipose tissue was down-regulated in the SHFD and restored in the PHFD to the level in the lean control mice. SIRT 3 was reported to be upregulated under conditions of caloric restriction (CR) and plays a role in regulating mitochondrial function. PNO consumption resulted in lower body fat and hepatic TG accumulation in HFD-induced obesity, which seemed to be associated with the CR-mimetic response.

## 1. Introduction

Obesity leads to a wide range of health problems and is associated with an increased risk of metabolic syndrome. Non-alcoholic fatty liver disease (NAFLD), a hepatic manifestation of metabolic syndrome, has been becoming a major public health problem due to the obesity epidemic [1]. It has been reported that one-third of the urban population in the United States had hepatic steatosis [2], and the prevalence of NAFLD in Asian countries has been reported to be 16%–18% [3,4]. NAFLD covers a spectrum of liver abnormalities ranging from simple steatosis to fibrosis or cirrhosis accompanied by severe inflammation. The mechanisms leading to hepatic lipid accumulation include increased lipid uptake, elevated *de novo* lipogenesis, improper fatty acid oxidation, and dysregulation of lipoprotein metabolism. An excessive level of hepatic lipids makes the liver vulnerable to further damage caused by inflammation [5]. There have been many efforts to discover a pharmacologic target for NAFLD treatment based on the pathological process of NAFLD; however, a strategy for treating NAFLD has not been firmly established [6]. Thus, lifestyle modifications focusing on improving an individual’s diet and level of physical activity have been proposed as a primary strategy for NAFLD treatment.

Dietary fatty acids play a significant role in modulating hepatic lipid metabolism [7]. Thus, not only the amount but also the composition of the dietary fat plays an important role in the prevention of NAFLD. It has been reported that saturated fatty acids contribute to the progression of hepatic steatosis by promoting endoplasmic reticulum stress and the apoptosis of hepatocytes [8], and conjugated linoleic acid (CLA) has also been linked to the development of hepatic steatosis despite its influence on weight reduction [9]. Conversely, omega-3 polyunsaturated fatty acids (PUFAs) have been known to have beneficial effects on hepatic lipid metabolism. The mechanisms behind these effects include reduced expression and/or activity of hepatic lipogenic genes, activation of hepatic fatty acid β-oxidation, and inhibition of the production of inflammatory mediators [10], which may result in protection from hepatic lipid accumulation. Recently, oil extracted from the seeds of *Pinus koraiensis*, pine nut oil (PNO), has been investigated for its impact on lipid metabolism. PNO contains pinolenic acid (18:3, Δ5, 9, 12), which is an unusual omega-6 PUFA characterized by polymethylene-interrupted double bonds. Asset *et al.* [11,12] showed that PNO consumption lowered the plasma cholesterol level compared with the consumption of other lipids such as sunflower oil, coconut oil, or lard in mice. Two previous studies have investigated PNO’s effects on weight-loss and NAFLD prevention. In a study by Ferramosca *et al.* [13], the consumption of a PNO-supplemented diet significantly reduced body weight gain and the liver weight relative to the consumption of a maize oil-supplemented diet in mice. In this study, a standard diet (12% of the energy from fat) was supplemented with PNO or maize oil, which made the diets have 30% of the energy from fat. However, a low-fat control group was not included in Ferramosca’s study, and the fat content of the diets was significantly lower than the amount in the commonly used high-fat diet (HFD) for diet-induced obesity. In another study by Ferramosca *et al.* [14], it was shown that the combination of CLA and PNO prevented a CLA-induced fatty liver and improved insulin sensitivity in mice. In this study, a standard diet (12% of the energy from fat) was supplemented with olive oil (control group), CLA and olive oil (CLA group), or CLA and PNO (CLA and PNO group) to make diets contain 30% of the energy from fat. Collectively, these findings suggest that PNO can affect weight gain and has the potential for NAFLD prevention by its modulation of lipid metabolism. However, the information regarding the role of PNO on NAFLD prevention is still limited. There is no study that has examined the effect of PNO in both a control diet and a HFD.

Some studies have demonstrated that PNO has appetite controlling effects [15,16], which suggested that PNO can lead to less energy consumption. Sirtuins (SIRTs), class 3 NAD^+^-dependent deacetylases, are known to be upregulated in the chronic caloric restriction (CR). It has been well-documented that SIRTs are responsible for the health benefits of CR such as longevity and protection from age-related diseases including cancer, neurodegeneration, and cardiovascular disease [17,18]. There are seven mammalian SIRTs, ranging from SIRT1 to SIRT7. Among the SIRTs, SIRT3 is the major mitochondrial form that has relevance in supporting fatty acid oxidation, enhancing the antioxidant defense system, and repairing mitochondrial DNA damage, which implies that SIRT3 can be a new target for protection against lipotoxicity and obesity-induced metabolic complications [19]. Thus, we examined whether the SIRT3 protein was upregulated in PNO-fed mice. Among the SIRTs, SIRT3 was chosen because of its significance not only in CR and but also in lipid metabolism as well.

In the present study, HFDs with 45% of the energy from fat with 10% from PNO or soybean oil (SBO) and 35% from lard were used to examine whether partial replacement with PNO in the HFD can ameliorate NAFLD in HFD-induced obese mice. The impact of PNO was also examined using control diets with 10% of the energy from fat from PNO or SBO. We measured the hepatic lipid contents and hepatic mRNA levels of genes involved in fatty acid oxidation and lipogenic pathways to evaluate the influence of PNO on hepatic fatty acid metabolism. Expression of the SIRT3 protein was measured in order to determine whether PNO had a similar effect to that of caloric restriction.

## 2. Materials and Methods

### 2.1. Animals and Diets

Five-week-old male C57BL/6 mice (Central Lab, Animal Inc., Seoul, Korea) were randomly divided into four groups after 3 days of acclimation. Mice were fed the experimental diets for 12 weeks *ad libitum*. Control diets contained 10% of the energy from fat from PNO (PC, *n* = 11) or SBO (SC, *n* = 10). HFDs contained 45% of the energy from fat with 35% from lard and 10% from PNO (PHFD, *n* = 11) or SBO (SHFD, *n* = 11). The PNO was a gift from the Dubio Co., Ltd. (Gyeonggi-do, Korea). The compositions of the experimental diets are shown in Table 1. The mice were housed individually and maintained in an animal facility with controlled temperature (23 ± 3 °C), controlled humidity (55% ± 10%), and a 12 h light/12 h dark cycle. SBO was chosen as the control oil because its fatty acid composition was similar to that of PNO except for its pinolenic acid content. The fatty acid compositions of the experimental diets are shown in Table 2. An antioxidant was added to the diets (0.2 μg of t-butylhydroquinone/g oil) to prevent the oxidation of polyunsaturated fatty acids, and fresh food was provided every other day. The body weights of the mice were recorded once a week, and their food intake was measured four times a week. At the end of the experimental period, the mice were fasted for 12 h and euthanized by asphyxiation with CO_2_. Blood was obtained by heart puncture, and serum was collected after centrifugation and stored at −80 °C for later analysis. White adipose tissue (epididymal, abdominal subcutaneous, and retroperitoneal-perirenal depots) was collected and stored at −80 °C for later analysis. For liver histology, fresh liver tissue was dissected into pieces in a consistent manner and fixed in 10% buffered neutral formalin. The rest of the liver tissue was stored at −80 °C for later analysis. This study was approved by the Animal Care and Use Committee at Seoul National University (approval No. SNU-101029-1).

**Table 1:** Composition of the experimental diets ^1^.

	Control Diet (g) (10% of the kcal from Fat)	High-Fat Diet (g) (45% of the kcal from Fat)
	10% Oil	10% Oil + 35% Lard
Casein	200	200
l-Cystine	3	3
Sucrose	350	172.8
Cornstarch	315	72.8
Dyetrose	35	100
PNO ^2^ or SBO	45	45
Lard	0	157.5
t-Butylhydroquinone	0.009	0.009
Cellulose	50	50
Mineral Mix ^3^	35	35
Vitamin Mix ^4^	10	10
Choline Bitartrate	2	2
Total	1045.0	848.1
kcal/g diet	3.69	4.64

^1^ Resource: Dyets, Inc, Bethlehem, PA, USA. ^2^ PNO was a gift from the Dubio Co., Ltd. ^3^ Thirty-five grams of mineral mix (Dyets, #210099) provides 1.0 g of sodium, 1.6 g of chloride, 0.5 g of magnesium, 0.33 g of sulfur, 59 mg of manganese, 45 mg of iron, 29 mg of zinc, 6 mg of copper, 2 mg of chromium, 1.6 mg of molybdenum, 0.16 mg of selenium, 0.9 mg of fluoride, 0.2 mg of iodine and 3.99 g of sucrose. ^4^ Ten grams of vitamin mix (Dyets, #300050) provides 4000 IU vitamin A, 1000 IU vitamin D_3_, 50 IU vitamin E, 30 mg of niacin, 16 mg of pantothenic acid, 7 mg of vitamin B_6_, 6 mg of vitamin B_1_, 6 mg of vitamin B_2_, 2 mg of folic acid, 0.5 mg of menadione, 0.2 mg of biotin, 10 µg of vitamin B_12_ and 9.78 g of sucrose.

**Table 2:** Fatty acid composition of the experimental diets (% of fatty acids) ^1^.

	SBO		PNO	
	Control Diet (SC)	High-Fat Diet (SHFD)	Control Diet (PC)	High-Fat Diet (PHFD)
Myristic acid (C14:0)	ND	0.9	ND	0.9
Palmitic acid (C16:0)	11.9	18.9	7.0	17.8
Stearic acid (C18:0)	4.8	11.1	3.6	10.7
Total saturated fatty acid	16.7	30.9	10.6	29.4
Palmitoleic acid (C16:1, Δ9)	ND	1.4	ND	1.4
Oleic acid (C18:1, Δ9)	21.1	34.7	27.4	36.0
Total monounsaturated fatty acid	21.1	36.1	27.4	37.4
Linoleic acid (C18:2, Δ9, 12)	54.9	30.3	47.2	28.6
α-linolenic acid (C18:3, Δ9, 12, 15)	7.4	2.8	0.8	1.3
Pinolenic acid (C18:3, Δ5, 9, 12)	ND	ND	14.0	3.3
Total polyunsaturated fatty acid	62.3	33.1	62.0	33.2

ND, not detected; ^1^ Fatty acid composition was determined by a gas chromatographic method.

### 2.2. Serum Lipid Concentrations

The serum non-esterified fatty acid (NEFA) (SICDIA NEFAZYME; Shin Yang Chemical, Seoul, Korea), triacylglycerol (TG) (Cleantech TG-S; Asan Pharmaceutical, Seoul, Korea), and total cholesterol (T-CHO; Asan Pharmaceutical, Seoul, Korea) concentrations were measured by enzymatic colorimetric methods according to the manufacturer’s instructions.

### 2.3. Serum Leptin and Fetuin-A Concentrations

Serum leptin (Quantikine^®^ ELISA kit; R & D Systems, Minneapolis, MN, USA) and fetuin-A (mouse Fetuin-A/AHSG Duo set; R & D Systems) levels were determined using enzyme-linked immunosorbent assays (ELISAs).

### 2.4. Liver Histology

For hematoxylin and eosin staining, liver tissue was embedded in paraffin and stained with hematoxylin and eosin. For oil red O staining, liver tissue was frozen in OCT compound and stained with oil red O. Histological images were captured at 200× magnification using a BX51 light microscope (Olympus, Optical Co. Ltd., Tokyo, Japan).

### 2.5. Hepatic Lipid Contents

The total lipids were extracted by the Folch method [20] from the liver tissue. The lipids were extracted in chloroform, dried in nitrogen gas and redissolved in isopropanol. The hepatic TG and cholesterol levels were determined by the same enzymatic colorimetric methods used for the measurement of the serum lipids. The hepatic TG and cholesterol contents are presented as mg/g tissue.

### 2.6. Real-Time PCR Analysis of the Genes in Liver Tissue

The mRNA levels of fetuin-A and genes involved in fatty acid oxidation and lipogenic pathways in the liver were determined. The oligonucleotide sequences of primers used in this study are presented in Table 3. The total RNA was isolated from the liver tissue using TRIzol reagent (Life Technologies Co., Carlsbad, CA, USA). The RNA concentration was measured by a spectrophotometer (Beckman Instruments, Fullerton, CA, USA), and the quality of the RNA was confirmed by agarose gel electrophoresis. The total RNA (2 μg) was used to synthesize single-stranded cDNA using a cDNA synthesis kit (PrimeScript™ II 1st strand cDNA synthesis kit; Takara Bio Inc., Shiga, Japan). The relative mRNA level was determined by the real-time quantitative PCR analysis using the SYBR green assay (SYBR^®^ Premix Ex TaqTM; Takara Bio Inc., Shiga, Japan), and the analysis was performed with a Step-One-Plus RT-PCR system (Life Technologies, Carlsbad, CA, USA). All of the samples were analyzed in duplicate with an endogenous control gene being analyzed at the same time. The relative expression levels of each gene were calculated by the 2^−ΔΔCT^ method, with the *Gapdh* gene being used as the endogenous control.

**Table 3:** Primer sequences used in real-time PCR quantitative analysis.

Gene	Function	Forward Primer (5′–3′)	Reverse Primer (5′–3′)
*Ahsg/*fetuin-A	fatty liver indicator	TTGCTCAGCTCTGGGGCT	GGCAAGTGGTCTCCAGTGTG
*Ppara*	transcription factor promoting fatty acid oxidation	GCAGTGGAAGAATCGGACCT	CAACCCGCCTTTTGTCATAC
*Cpt1a*	mitochondrial β-oxidation	GATGTTCTTCGTCTGGCTTGA	CTTATCGTGGTGGTGGGTGT
*Acadl*	mitochondrial β-oxidation	TCGCAATATAGGGCATGACA	ACTTGGGAAGAGCAAGCGTA
*Hadha*	mitochondrial β-oxidation	CCCTTTGAACACTTGCTGCT	GCCCAGGTCTCTGTGGATAA
*Acox1*	peroxisomal β-oxidation	GTCAAAGGCATCCACCAAAG	GAGGGGAACATCATCACAGG
*Cyp4a10*	microsomal ω-oxidation	CAGAAAGGAGGGAAGATGGAG	CATGGTCTCCAAAATCCAAGG
*Sod2*	anti-oxidative defense	TTAGAGCAGGCAGCAATCTGT	GCGTGACTTTGGGTCTTTTG
*Ucp2*	anti-oxidative defense	CAGGTCACTGTGCCCTTACCA	CACTACGTTCCAGGATCCCAA
*Pparg*	transcription factor promoting adipogenesis	CAGCAGGTTGTCTTGGATGTC	AGCCCTTTGGTGACTTTATGG
*Srebf1*	*de novo* lipogenesis	GTCTCCACCACTTCGGGTTT	CGACTACATCCGCTTCTTGC
*Fasn*	*de novo* lipogenesis	GCGGTGTGAAAACGAACTTT	CTGTCTGGGCATAACGGTCT
*Slc25a1*	*de novo* lipogenesis	TTCCCTTTAGCCCTTGTTCC	TGACCAGACTTCCTCCAACC
*Fabp1*	fatty acid transport	GAACTCATTGCGGACCACTT	CATCCAGAAAGGGAAGGACAT
*Cd36*	fatty acid transport	CCAAGCTATTGCGACATGATT	TCTCAATGTCCGAGACTTTTCA
*Gapdh*	endogenous control	GGAGAAACCTGCCAAGTA	AAGAGTGGGAGTTGCTGTTG

*Ahsg/*fetuin-A, alpha-2-HS-glycoprotein; *Ppara*, peroxisome proliferator activated receptor alpha; *Cpt1a*, carnitine palmitoyltransferase 1a; *Acadl*, long-chain acyl-CoA dehydrogenase; *Acox1*, acyl-CoA oxidase 1; *Cyp4a10*, cytochrome P450 family 4 subfamily a polypeptide 10; *Hadha*, hydroxyacyl-CoA dehydrogenase alpha subunit; *Sod2*, superoxide dismutase 2; *Ucp2*, uncoupling protein 2; *Pparg*, peroxisome proliferator activated receptor gamma; *Srebf1*, sterol regulatory element-binding transcription factor 1; *Fasn*, fatty acid synthase; *Slc25a1*, solute carrier family 25 member 1; *Fabp1*, fatty acid binding protein 1; *Cd36*, cluster of differentiation 36; *Gapdh*, glyceraldehyde-3-phosphate dehydrogenase.

### 2.7. Western Blot Analysis of SIRT3 in White Adipose Tissue

The epididymal fat pad was homogenized in an ice-cold protein lysis buffer containing 50 mM Tris-Cl (pH 7.4), 150 mM NaCl, 1 mM EDTA, 1 mM PMSF, 1 mM Na_3_VO_4_, 1 mM NaF, 1 mM Na_4_P_2_O_7_, 1 mM β-glycerophosphate, 1% NP-40, 0.25% sodium deoxycholate, 10% glycerol, and protease inhibitor cocktail (Complete Mini Protease Inhibitor Cocktail; Roche Diagnostics GmbH, Penzberg, Germany). The supernatant was obtained by centrifugation at 10,000× *g* for 30 min at 4 °C. A Bradford assay (Bio-Rad, Hercules, CA, USA) was used to determine the total protein concentration. Fifty micrograms of the protein sample was separated in 10% SDS-PAGE and transferred onto a PVDF membrane (Bio-Rad, Hercules, CA, USA). The membrane was blocked with 3% bovine serum albumin (BSA) in Tris-buffered saline containing 0.1% Tween-20 (pH 7.6, TBST) for 1 h. The membrane was incubated with rabbit anti-mouse SIRT3 antibody (1:1000; Cell Signaling Technology, Beverly, MA, USA) in 3% BSA/TBST overnight at 4 °C. The membrane was washed with TBST and then incubated with secondary anti-rabbit IgG HRP-linked antibody (Cell Signaling Technology, Beverly, MA, USA) in 3% BSA/TBST for 1 h at room temperature. The signal was visualized by a chemiluminescent detection system and quantified using Quantity One analysis software (Bio-Rad, Hercules, CA, USA).

### 2.8. Statistical Analysis

Statistical analysis was performed using PASW Statistics 18 (SPSS Inc., Chicago, IL, USA). Two-way ANOVA was used to evaluate the overall effects of the fat amount, oil type, and their interaction. When the effects of the fat amount and/or oil type were significant, a LSD multiple-comparison post-hoc test was performed for the individual group comparisons. Student’s *t* test was used for the comparison between the PC and SC groups or the PHFD and SHFD groups if an interaction was significant. Pearson’s correlation was used to determine the relationship between the two variables. The results from all of the comparisons were considered significant at *p* < 0.05. Data are reported as the means ± SEM.

## 3. Results

### 3.1. Body Weight, Energy Intake, and Body Fat Accumulation

After 12 weeks of feeding, HFD-fed mice had a significantly higher mean body weight (*p* < 0.05) and a significantly higher mean white adipose tissue weight (*p* < 0.05) than control diet-fed mice. Mice in the PHFD group gained less body weight (10% less, *p* < 0.05) and had less white adipose tissue (18% less, *p* < 0.05) compared to mice in the SHFD group, but their energy intake was not significantly different from that of mice in the SHFD group. Mice in the PC group also had less white adipose tissue than mice in the SC group (30% less, *p* = 0.05) (Table 4). Body weight gain positively correlated with the white adipose tissue weight (*r* = 0.93, *p* < 0.05), which indicated that the PNO-mediated lower weight gain was mainly due to a lower body fat accumulation. Mice in the PHFD group also had a significantly lower serum leptin level than those in the SHFD group (*p* < 0.05), which was consistent with the result that PNO suppressed body fat accumulation in the HFD-induced obese mice (Table 5).

**Table 4:** Body weights, energy intakes, and adipose tissue weights.

	SC (*n* = 10)	PC (*n* = 11)	SHFD (*n* = 11)	PHFD (*n* = 11)	*p* Value		
					Fat Amount	Oil Type	Interaction
Body weight at 0 week (g)	17.3 ± 0.5	16.7 ± 0.4	17.0 ± 0.4	17.0 ± 0.3	0.97	0.56	0.50
Body weight at 12 weeks (g)	32.8 ± 1.0 ^ab^	30.5 ± 0.6 ^a^	38.5 ± 1.4 ^c^	34.6 ± 1.4 ^b^	0.00	0.01	0.49
Weight gain (g)	15.5 ± 0.8 ^ab^	13.8 ± 0.6 ^a^	21.5 ± 1.1 ^c^	17.5 ± 1.3 ^b^	0.00	0.01	0.32
Average daily food intake (g/day)	3.20 ± 0.06 ^b^	3.20 ± 0.03 ^b^	2.82 ± 0.05 ^a^	2.76 ± 0.04 ^a^	0.00	0.54	0.48
Average daily energy intake (kcal/day) ^1^	11.8 ± 0.2 ^a^	11.8 ± 0.1 ^a^	13.1 ± 0.2 ^b^	12.8 ± 0.2 ^b^	0.00	0.50	0.43
Total white adipose tissue (g) ^2^	3.1 ± 0.2 ^b^	2.2 ± 0.2 ^a^	5.3 ± 0.4 ^d^	4.4 ± 0.4 ^c^	0.00	0.00	0.95
Epididymal (g)	1.3 ± 0.1 ^b^	0.9 ± 0.1 ^a^	2.2 ± 0.2 ^c^	1.9 ± 0.2 ^c^	0.00	0.00	0.68
Retroperitoneal and perirenal (g)	0.60 ± 0.04 ^b^	0.41 ± 0.05 ^a^	0.99 ± 0.06 ^c^	0.84 ± 0.08 ^c^	0.00	0.01	0.79
Abdominal subcutaneous (g)	1.2 ± 0.1 ^a^	0.9 ± 0.1 ^a^	2.1 ± 0.2 ^c^	1.7 ± 0.2 ^b^	0.00	0.01	0.52

Values are presented as the mean ± SE. Two-way ANOVA was used to determine the significant effects of the fat amount and oil type and was followed by a LSD post-hoc test. Means in a row without a common superscript are significantly different (*p* < 0.05). ^1^ Average daily energy intake (kcal/day) = Average daily food intake (g/day) × Energy per g diet (kcal/g diet); ^2^ WAT weight is the sum of the weights of the epididymal, abdominal subcutaneous, and retroperitoneal-perirenal depots.

### 3.2. Serum Lipid Levels

PNO tended to decrease the total cholesterol in the serum (*p* = 0.09), whereas it had no significant influence on the TG concentration of the serum. Serum NEFA, the primary factor that causes hepatic lipid overload in obesity, tended to be at a higher level in HFD-fed mice than in control diet-fed mice (*p* = 0.09), but no significant effect of PNO was observed (Table 5).

**Table 5:** Serum lipid and leptin levels.

	SC (*n* = 10)	PC (*n* = 11)	SHFD (*n* = 11)	PHFD (*n* = 11)	*p* value		
					Fat Amount	Oil Type	Interaction
Serum triacylglycerol (mg/dL)	116.0 ± 8.4	133.5 ± 10.4	163.0 ± 33.8	118.1 ± 16.1	0.44	0.50	0.13
Serum cholesterol (mg/dL)	282.8 ± 10.5	250.2 ± 15.7	284.7 ± 22.1	258.1 ± 16.2	0.77	0.09	0.86
Serum NEFA (mmol/L)	1.09 ± 0.08	1.35 ± 0.08	1.71 ± 0.40	1.62 ± 0.29	0.09	0.73	0.50
Serum leptin (ng/mL)	19.9 ± 2.8 ^ab^	12.6 ± 2.0 ^a^	43.3 ± 6.8 ^c^	29.2 ± 4.5 ^b^	<0.01	0.02	0.46

Values are presented as the mean ± SE. Two-way ANOVA was used to determine the significant effects of the fat amount and oil type and was followed by a LSD post-hoc test. Means in a row without a common superscript are significantly different (*p* < 0.05).

### 3.3. Liver Lipid Levels

**Figure 1:**
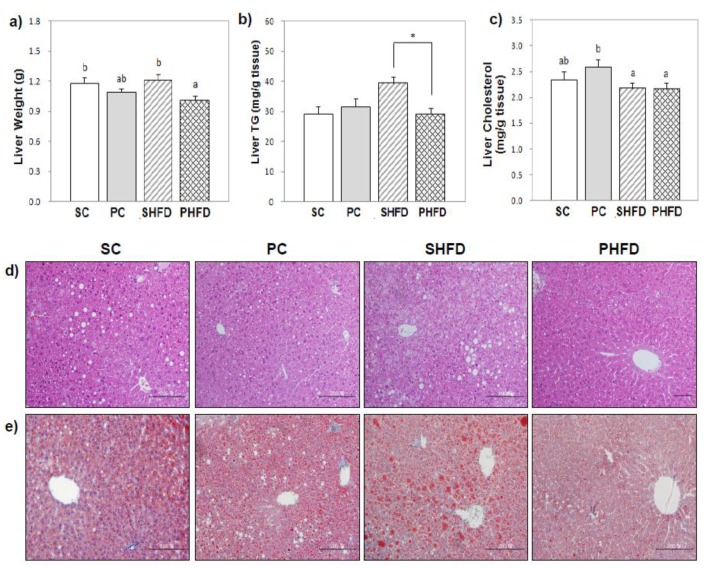
Liver weight and liver lipid levels (**a**) liver weight; (**b**) liver triacylglycerol (TG); (**c**) liver cholesterol; (**d**) representative photomicrographs of liver sections stained with hematoxylin and eosin (×200), and (**e**) representative photomicrographs of liver sections stained with Oil red O (×200). Values are the mean ± SE (SC, *n* = 10; PC, *n* = 11; SHFD, *n* = 11; PHFD, *n* = 11). Two-way ANOVA was used to determine the effects of the fat amount and oil type and was followed by a LSD post-hoc test. Labeled means without a common superscript represent a significant difference at *p* < 0.05. Student’s *t* test was used for comparison between the PC and SC groups or the PHFD and SHFD groups if the interaction was significant. * indicates a significant difference at *p* < 0.05.

The mean liver weight of mice in the PHFD group was lower than that of the SHFD group (9% less, *p* < 0.05) (Figure 1a). For the hepatic TG concentration, there was a significant interaction between the amount of fat and the oil type (*p* < 0.05). The hepatic TG concentration of the PHFD group was significantly lower than that of the SHFD group (26% less, *p* < 0.05), and it was comparable to that of lean mice (Figure 1b). When the total hepatic TG content was calculated and adjusted for body weight, it confirmed that mice in the PHFD group stored less TG in the liver compared to those in the SHFD group (24% less, *p* < 0.05). There was no significant difference in the hepatic TG concentration between the SC and PC groups. PNO had no significant influence on the hepatic cholesterol level. Overall, the hepatic cholesterol level was lower in HFD-fed mice than in control diet-fed mice (12% lower, *p* < 0.05), which was mainly due to the high cholesterol level of the PC group (Figure 1c).

### 3.4. Hepatic Fetuin-A mRNA and Serum Fetuin-A Levels

**Figure 2:**
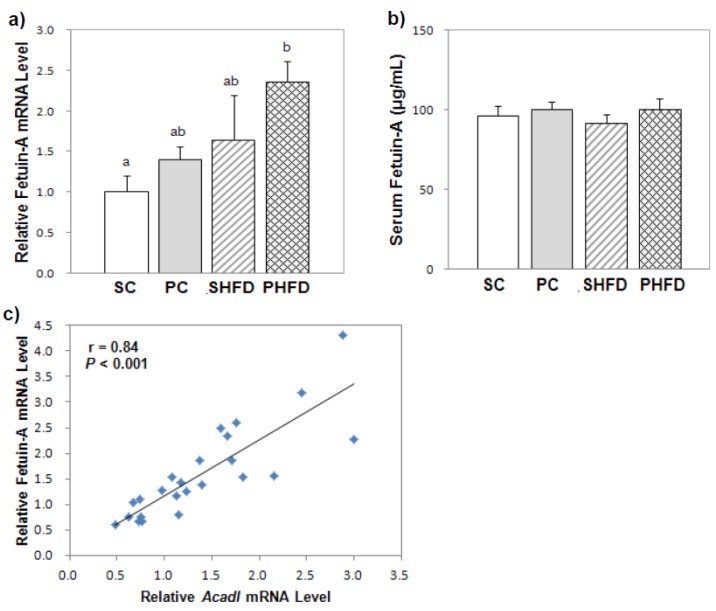
Hepatic fetuin-A mRNA and serum fetuin-A levels (**a**) hepatic fetuin-A mRNA level; (**b**) serum fetuin-A level; (**c**) correlation between hepatic fetuin-A mRNA and *Acadl* mRNA levels. The serum fetuin-A level was measured by ELISA (SC, *n* = 10; PC, *n* = 11; SHFD, *n* = 11; PHFD, *n* = 11). The mRNA level was determined by a real-time PCR method (*n* = 6 per group). Values are the mean ± SE. Two-way ANOVA was used to determine the effects of the fat amount and oil type and was followed by a LSD post-hoc test. Labeled means without a common superscript represent a significant difference (*p* < 0.05).

To examine whether NAFLD was developed due to a HFD and whether PNO had a beneficial influence for the prevention of NAFLD, hepatic fetuin-A mRNA and serum fetuin-A levels were measured. Although the fetuin-A mRNA level was significantly higher in obese mice (1.7-fold, *p* < 0.05), the serum fetuin-A level was not significantly higher in obese mice compared to the level in lean mice in this study (Figure 2a,b). Overall, PNO consumption tended to result in higher fetuin-A mRNA levels in the liver (1.4-fold, *p* = 0.1) even though PNO-fed mice did not show steatotic livers. Fetuin-A mRNA levels positively correlated with the mRNA levels of *Acadl*, an enzyme catalyzing the first step of mitochondrial β-oxidation of fatty acids (*r* = 0.84, *p* < 0.05) (Figure 2c), and with the mRNA levels of other genes involved in fatty acid β-oxidation (*Ppara*, *r* = 0.56, *p* < 0.05; *Hadha*, *r* = 0.70, *p* < 0.05; *Acox1*, *r* = 0.49, *p* < 0.05).

### 3.5. Hepatic Expression of Genes Involved in Fatty Acid Oxidation and Oxidative Stress

**Figure 3:**
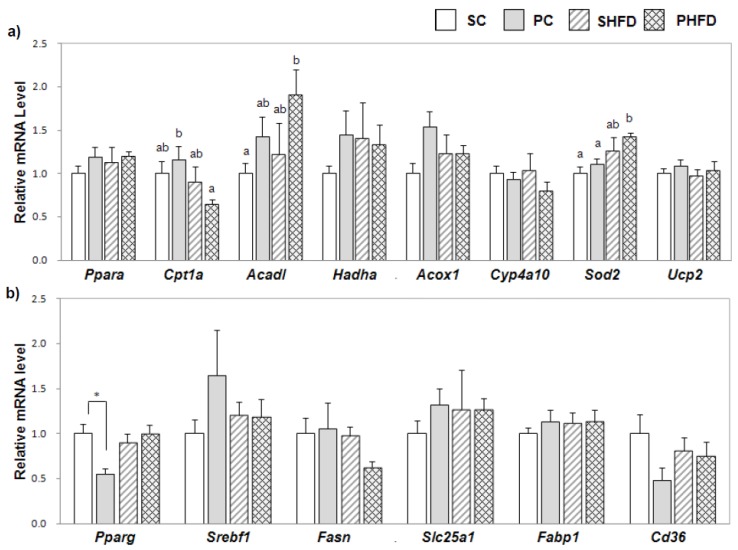
Expression of hepatic genes involved in fatty acid oxidation, oxidative stress, and lipogenic pathways (**a**) hepatic mRNA levels of genes involved in fatty acid oxidation and oxidative stress and (**b**) hepatic mRNA levels of genes involved in lipogenic pathways. The mRNA level was determined by a real-time PCR method. Values are the mean ± SE (*n* = 6 per group). Two-way ANOVA was used to determine the effects of the fat amount and oil type and was followed by a LSD post-hoc test. Labeled means without a common superscript represent a significant difference at *p* < 0.05. Student’s *t* test was used for comparison between the PC and SC groups or the PHFD and SHFD groups if the interaction was significant. * indicates a significant difference at *p* < 0.05.

We examined the expression of genes involved in fatty acid oxidation to see whether the PNO-mediated decrease in hepatic TG accumulation in obese mice could be attributed to enhanced fatty acid oxidation (Figure 3a). Overall, PNO consumption promoted mRNA expression of *Acadl* (1.5-fold, *p* = 0.05). Mice in the PHFD group tended to have higher *Acadl* mRNA levels compared to mice in the SHFD group (1.6-fold, *p* = 0.08). The mRNA levels of *Sod2*, an anti-oxidative enzyme, were higher in HFD-fed mice (1.3-fold, *p* < 0.05), and the PHFD group showed the highest level. The mRNA level of *Cpt1a*, a rate-limiting enzyme that transfers fatty acids from the cytosol into the mitochondria, was lower in HFD-fed mice (0.7-fold, *p* < 0.05), and the PHFD group had the lowest level of *Cpt1a* mRNA among the four groups. The mRNA expression levels of *Ppara*, *Acox1*, *Hadha*, *Cyp4a10*, and *Ucp2* were not significantly affected by HFD or PNO.

### 3.6. Hepatic Expression of Genes Involved in Lipogenic Pathways

To examine the effect of PNO on lipogenic pathways in the liver, we examined the expression of lipogenic genes in the liver (Figure 3b). Although the level of *Pparg* mRNA was not greater in obese mice, it was significantly lower in the PC group compared to in the SC group (0.5-fold, *p* < 0.05). The mRNA levels of *Srebf1*, *Fasn*, *Slc25a1*, *Fabp1*, and *Cd36* were not significantly altered by HFD or PNO.

### 3.7. SIRT3 Protein Expression in White Adipose Tissue

**Figure 4:**
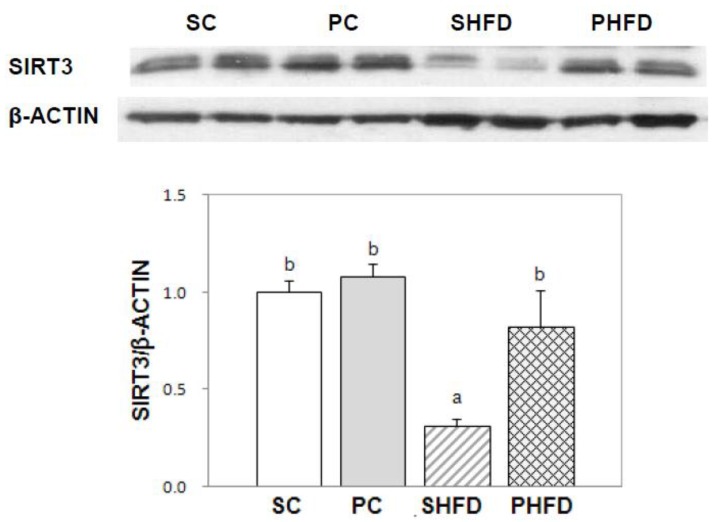
SIRT3 protein expression in white adipose tissue. Epididymal adipose tissue protein (50 μg) was used for western blot analysis. A representative image is presented. The intensity of the SIRT3 band was densitometrically measured and normalized to the level of β-Actin. Values are the mean ± SE (SC, *n* = 5; PC, *n* = 5; SHFD, *n* = 6; PHFD, *n* = 6). Two-way ANOVA was used to determine the effects of the fat amount and oil type and was followed by a LSD post-hoc test. Labeled means without a common superscript represent a significant difference at *p* < 0.05.

We examined whether the expression of SIRT, a protein known to be responsible for CR-mediated health benefits, was higher in the white adipose tissue of PNO-fed mice (Figure 4). The protein expression of SIRT3 was lowered by being fed a high-fat diet (*p* < 0.05), and it was restored in the PHFD group to the level in lean mice. The PHFD group had a significantly higher expression of the SIRT3 protein compared to the SHFD group (*p* < 0.05)

## 4. Discussion

In this study, we showed that hepatic TG accumulation was attenuated by PNO replacement in HFD-fed mice. Mice in the SHFD group showed 30% higher hepatic TG concentrations compared to the control group, but mice in the PHFD group maintained hepatic TG concentrations at the level of lean mice. NAFLD induced by feeding the mice a HFD (45% of the energy from fat) for 12 weeks in this study seemed to remain in the initial stage of a simple steatotic condition. A hepatic TG content over 5% of the liver weight, which is the criteria for a fatty liver [21], was not observed overall. On pathological observation, inflammation and hepatocyte degeneration were mild (Table A1) even in the SHFD group. The expression of *Pparg* mRNA was also not higher in the HFD-fed mice compared with the control diet-fed mice. Meanwhile, the hepatic cholesterol level was lower in HFD-fed mice, and PNO had no effect on the hepatic cholesterol level. In the study by de Vogel-van den Bosch *et al.* [22], feeding a cholesterol-free high-fat diet lowered fractional cholesterol absorption, which resulted in cholesterol synthesis and down-regulation of cholesterol efflux to spare intracellular cholesterol for chylomicron formation. Desmarchelier *et al.* [23] also reported that mice fed with the high-fat western diet containing 0.03% (*w*/*w*) cholesterol showed reduced intestinal and hepatic cholesterol concentrations despite the hypercholesterolemia and increased hepatic TG levels. The HFD used in this study provided 0.02% (*w*/*w*) cholesterol from lard. It seems that providing this amount of cholesterol is not sufficient to meet the increased demand for chylomicron packaging due to lipid overload with high-fat diet feeding. The duration of high-fat feeding was not very long in this study (12 weeks) and two studies by others (8 weeks in the study by de Vogel-van den Bosch *et al.* [22] and 12 weeks in the study by Desmarchelier *et al.* [23]). Taken together, lower levels of hepatic cholesterol in HFD fed mice in this study might be interpreted as an adaptive response to deal with large quantities of fat in the diet.

The mRNA level of long-chain acyl-CoA dehydrogenase *(Acadl)* in PNO-fed mice was significantly higher, and the PHFD group expressed the highest level of *Acadl* among the four groups. The up-regulation of *Acadl* mRNA might contribute to the enhancement of fatty acid β-oxidation, which could partially explain the smaller hepatic TG accumulation in PNO-fed mice. The role of fatty acid oxidation in NAFLD is controversial. Studies by Schrauwen *et al.* [24] and Sunny *et al.* [25] reported that fatty acid oxidation was higher in steatotic livers, which could lead to oxidative stress and liver damage. However, the increase in fatty acid oxidation in both studies was attributed to the excessive amount of TG stored in the liver tissue, whereas PNO-fed mice in this study showed little TG accumulation. Additionally, the PHFD group had the highest level of superoxide dismutase 2 (*Sod2)* mRNA among the four groups, which might confer a protection from the reactive oxygen species produced by increased fatty acid oxidation. Because *Sod2* can also be induced by reactive oxygen species [26], this result is consistent with the fact that the PHFD group showed increased expression of *Acadl*. However, the PFHD group had the lowest mRNA expression level of carnitine palmitoyltransferase 1a *(Cpt1a)* among the four groups. Depletion of unesterified coenzyme A, a cofactor required for fatty acid oxidation in the mitochondria, would further inhibit the entry of fatty acids into the mitochondria [27,28]. As increased mitochondrial β-oxidation could lead to a shortage of available cofactors, down-regulation of *Cpt1a* mRNA in the PHFD group might be the result of an intramitochondrial control mechanism to prevent a further influx of fatty acids in order to maintain mitochondrial homeostasis. The results from a recent study by Le *et al.* [29], in which PNO increased both *Cpt1b* and *Acadl* mRNA levels in the muscle tissue of HFD-fed mice, suggested a tissue-specific regulation. In fact, liver and muscle tissues express different isoforms of *Cpt1*, and the two isoforms have different kinetic characteristics [28]. However, direct mechanistic evidence to explain the contradictory expression pattern of the genes involved in mitochondrial β-oxidation (higher *Acadl* and lower *Cpt1*) shown in this study is lacking. A direct comparison of the mitochondrial fatty acid oxidation rates would help confirm the effect of PNO on fatty acid oxidation in the liver.

Fetuin-A has been linked to weight gain, insulin resistance, and NAFLD [30,31,32]. Fetuin-A knockout mice were insulin-sensitive and resistant to weight gain when fed a HFD [33]. Stefan *et al.* [34] reported that the plasma fetuin-A level positively correlated with liver fat accumulation in humans, and the expression of fetuin-A mRNA was greater in mice with fatty livers. In this study, the serum fetuin-A concentration was not higher in obese mice despite the upregulated hepatic fetuin-A mRNA level in obese mice. Because fetuin-A is present in systemic circulation at a high concentration, the magnitude of the elevated fetuin-A expression in the liver might have been insufficient to cause a significant change in the serum fetuin-A concentration. However, the hepatic expression of fetuin-A mRNA was greater in PNO-fed mice without evidence of hepatic steatosis, and it positively correlated with the genes involved in fatty acid oxidation. Haukeland *et al.* [35] reported that hepatic fetuin-A mRNA levels positively correlated with *Pepck1* and *Srebp1c* mRNA levels in human subjects. A positive correlation between fetuin-A and *Cpt1* mRNA levels was also observed, although it was less consistent. These results suggest that fetuin-A mRNA expression could be co-regulated with glucose and lipid metabolism. Based on our results, the expression of fetuin-A mRNA seemed to be closely related to fatty acid oxidative metabolism, and the up-regulation of fetuin-A mRNA in PNO-fed mice could be the result of increased fatty acid oxidation. Further studies are needed to elucidate the relationship between fetuin-A and fatty acid metabolism.

Although the overall effect of PNO on the mRNA expression of fatty acid synthase *(Fasn)*, a multi-enzyme protein involved in *de novo* lipogenesis, was not significant, it was significantly lower in the PHFD group than in the SHFD group (Student’s *t*-test, *p* < 0.05). Ferramosca *et al.* [14] reported that the activities of hepatic lipogenic enzymes including acetyl-CoA carboxylase and fatty acid synthase were significantly decreased in PNO-fed mice after 16 weeks of feeding, and PNO-fed mice were protected from liver fat accumulation when CLA was fed, which partially supports our results. In the study by Donnelly *et al.* [36], *de novo* lipogenesis was elevated in the fasting state and failed to increase in the postprandial state in NAFLD patients, which suggested dysregulated *de novo* lipogenesis in NAFLD patients. Our results indicated that the attenuation of the lipogenic process under the condition of HFD consumption occurred in mice in the PHFD group, while this was not observed in mice in the SHFD group. Collectively, these data suggested that PNO replacement contributes to the maintenance of the homeostatic regulation of hepatic fatty acid metabolism in HFD-induced obesity by increasing fatty acid β-oxidation and alleviating lipogenesis.

The changes in the expression of hepatic genes related to fatty acid oxidation and lipogenesis by PNO replacement have been discussed so far. In this study, PNO-fed mice showed reduced weight gain and body fat accumulation along with the less hepatic TG accumulation in the HFD group. It is well established that an excess flow of free fatty acids arising from adipose tissue is the major contributor to hepatic TG in obese patients [36], which is mainly explained by the failed suppression of lipolysis caused by peripheral insulin resistance. Thus, there is a possibility that less hepatic TG accumulation in the PHFD group in this study could be an indirect consequence of less body fat accumulation followed by an overall improvement in energy metabolism.

Among the SIRTs (SIRT1-7), SIRT3 plays a pivotal role in energy metabolism and mitochondrial function by regulating the activity of mitochondrial enzymes related to fatty acid metabolism including ACADL [37] and SOD2 [38]. We observed that SIRT3 protein expression was significantly lower in the white adipose tissue of the SHFD group compared with that of mice fed control diets, which seemed to be the result of excess energy storage in the white adipose tissue of mice in the SHFD group. Conversely, SIRT3 protein expression in the white adipose tissue from the PHFD group was maintained at the same level as that of the control diet-fed mice and was not reduced despite the HFD feeding. These results suggested that partial PNO replacement prevented the down-regulation of SIRT3 caused by high-fat feeding and that PNO could alleviate mitochondrial dysregulation by maintaining SIRT3 protein expression in HFD-induced obesity.

Collectively, it seemed that PNO-fed mice showed mild CR-mimetic responses in both the control diet and HFD groups, although the actual energy intake was not significantly different from the corresponding SBO-containing diet groups. First, mice in the PHFD group showed less hepatic lipid accumulation accompanied with less whole-body fat accumulation compared with the mice in the SHFD group. Hepatic lipid reduction with whole-body weight loss is commonly observed when obese animals [39] or human subjects [40,41] were placed on CR. Higher SIRT3 protein expression in white adipose tissue from the PHFD group than that from the SHFD group further supports this idea. Secondly, mice in the PC group also showed the least body weight gain among the four groups and also showed markedly reduced *Pparg* mRNA expression compared to the other groups. It was reported that the hepatic *Pparg* mRNA level was higher in NAFLD patients [42], and CR reduced the expression of hepatic *Pparg* mRNA in mice [43]. Thirdly, the serum NEFA levels of mice in the PC group were higher than those of mice in the SC group (Student’s *t*-test, *p* < 0.05) even though mice in the PC group had less white adipose tissue than those in the SC group. Elevation of fasting serum NEFA is a metabolic feature of long-term CR [44], which is a result of enhanced fat mobilization as an adaptive response to CR.

The pinolenic acid (18:3, Δ5, 9, 12) in PNO (14% of the fatty acid composition) is a strong candidate for the cause of the PNO-mediated effect observed in this study. In the study by Le *et al.* [29], pinolenic acid elicited ligand activity for PPARα and PPARδ, and pinolenic acid treatment upregulated the downstream target genes of PPARα and PPARδ involved in fatty acid oxidation, including *Pgc1a*, *Ucp3*, *Cpt1b*, *Acadm*, and *Acadl* in the C2C12 myotubes cell line. The ligand activity of pinolenic acid for PPARα and PPARδ could be a reason for the lower body fat accumulation in PNO-fed mice considering the importance of PPAR in the lipid metabolism of adipose tissue. However, Le *et al.* mainly focused on skeletal muscle and brown adipose tissue. Thus, mechanistic studies that examine the expression of downstream genes of PPARα and PPARδ using liver and white adipose tissue cell lines could be helpful for achieving further understanding.

## 5. Conclusions

In conclusion, PNO consumption resulted in less body fat accumulation. Partial PNO replacement in the HFD ameliorated hepatic steatosis and prevented the down-regulation of SIRT3 in white adipose tissue. It seemed that PNO exerted a CR-mimetic effect despite no significant change in the food intake, which prevented a dysregulation of energy homeostasis in HFD-induced obesity.

## Appendix

**Table A1:** NAFLD activity scores of mice fed control diets or HFDs containing SBO or PNO.

	SC (*n* = 6)	PC (*n* = 7)	SHFD (*n* = 7)	PHFD (*n* = 7)	*p* Value		
					Fat Amount	Oil Type	Interaction
Steatosis	1.00 ± 0.26	0.71 ± 0.29	1.00 ± 0.31	0.86 ± 0.26	0.80	0.46	0.80
Ballooning	0.50 ± 0.34	0.57 ± 0.37	1.00 ± 0.38	0.86 ± 0.34	0.29	0.92	0.77
Inflammation	0.50 ± 0.22	0.14 ± 0.14	0.29 ± 0.18	0.14 ± 0.14	0.54	0.16	0.54
NAFLD activity score (NAS)	2.00 ± 0.52	1.43 ± 0.48	2.29 ± 0.64	1.86 ± 0.63	0.54	0.40	0.90

Hematoxylin and eosin (H & E) staining was used for evaluation of steatosis, degeneration of hepatocyte, and inflammation and accumulation of fat droplet was also confirmed by oil red O staining. Steatosis, hepatocellular ballooning, and lobular inflammation were graded as described previously [45] with modification. The degree of steatosis was graded using on a scale: grade 0, minimal; grade 1, moderate; grade 2, severe. Hepatocellular ballooning graded using on a scale: grade 0, minimal; grade 1, a few balloon cells; grade 2, many/prominent balloon cells. Lobular inflammation was graded on a scale: grade 0, fewer than two foci per ×20 field; grade 1, two to four foci per ×20 field; grade 2, more than four foci per ×20 field. For the NAFLD activity score (NAS), grades of steatosis, hepatocellular ballooning, and lobular inflammation were combined, ranging from 0 to 6.
